# Climate Change and Veterinary Medicine: A Call to Action for a Healthier Planet

**DOI:** 10.12688/f1000research.158307.1

**Published:** 2024-11-13

**Authors:** Winnifred Akello

**Affiliations:** 1 College of Veterinary Medicine, Animal Resources and Biosecurity, Makerere University, Kampala, Uganda

**Keywords:** Climate Change, Veterinarians, One Health, Climate-Resilience, Sustainable Agriculture, Biodiversity, Public Health, Climate Action

## Abstract

Climate change is rapidly transforming ecosystems and reshaping the landscapes of animal health, with profound consequences for public health, food security, and biodiversity. Rising temperatures, shifting weather patterns, and increased frequency of natural disasters are driving the emergence and spread of infectious diseases, particularly zoonotic and vector-borne diseases. These environmental shifts endanger the health and welfare of animals and the delicate balance between human populations, livestock, and wildlife. As the stewards of animal health, veterinarians are uniquely positioned to lead the change in addressing these complex challenges at the nexus of human, animal, and environmental health and well-being.

This article calls for urgent actions to integrate climate adaptation and mitigation strategies into veterinary practice and education. It underscores the critical need for veterinarians to embrace the One Health approach to tackle climate-driven disease outbreaks and the growing threat of antimicrobial resistance to safeguard human and animal populations while protecting natural ecosystems. The article further explores the role of veterinarians in fostering sustainable agricultural practices, reducing the environmental impact of livestock production, conserving biodiversity and advocating for policy reforms that protect both animal and planetary health.

As we face an era of unprecedented climate disruption, this call to action aims to inspire the global veterinary community to actively get involved in combating climate change and its worst impacts. By building climate-resilient practices, enhancing disease surveillance, and championing environmental stewardship, veterinarians can contribute significantly to a healthier, more sustainable future for all species on Earth.

## Introduction

In the face of one of the most pressing challenges of our time – climate change – the veterinary profession stands at a critical juncture. Often perceived solely as protectors of animal health, veterinarians now find themselves as frontline defenders of the environment, public health, and the fragile ecosystems that sustain all life. Climate change is not just an environmental issue; it is a public health emergency that deeply affects human, animal, and ecological well-being. It is time for the veterinary community to rise to the challenge, embrace a proactive role in combating climate change, and lead with the values of the One Health approach.

The connection between climate change and animal health is multi-dimensional. Animals, specifically livestock, are a significant factor in the greenhouse gas emissions associated with agriculture, contributing approximately 14.5% of all anthropogenic greenhouse gases globally ( FAO 2017). At the same time, climate change is threatening all life. Increasing temperatures, rising sea levels, frequent extreme weather events like floods, cyclones, droughts, and uncontrollable wildfires ( Dey and Lewis 2021) are the harrowing realities of a rapidly changing climate. These changes disrupt ecosystems, destroy habitats, shift disease patterns and drive unprecedented biodiversity loss ( Upadhyay 2020).

Veterinarians are already seeing the consequences: vector-borne diseases such as bluetongue virus and parasitic infections in livestock are spreading and becoming more prevalent in once-safe areas ( Lacetera 2019). Heat stress in livestock reduces productivity and increases disease vulnerability ( FAO 2020). Wild animals are migrating in search of cooler habitats, and many have become extinct ( Ziswiler 2012), while pets face new health challenges as their environments change ( Narayan 2023).

As stewards of animal health, veterinarians cannot watch as the climate crisis endangers the very species we have sworn to protect.

At the heart of veterinary medicine is the One Health principle, which emphasizes that human, animal, and environmental health are inextricably linked. Climate change is driving home this truth with brutal clarity. The rise in zoonotic diseases like avian influenza, Dengue and Rift Valley fever, the increased threat of food insecurity, and foodborne pathogens are direct consequences of climate-driven environmental disruptions ( Rupasinghe et al. 2022). Additionally, climate change has been linked to antimicrobial resistance because of the alarming spread of diseases, which results in the escalating use of antimicrobials ( Lio et al. 2023).

## Why must the veterinary professionals act?

Safeguarding animal health is safeguarding human health. The veterinary profession must become advocates for environmental stewardship, educators in sustainable practices, and innovators in mitigating the impacts of climate change on health. Here is why and how the veterinary professionals must act:

**Zoonotic disease prevention**: As climate change alters disease patterns and drives wildlife closer to domestic animals and human populations, veterinarians play a crucial role in early disease detection and response to outbreaks. By preventing zoonotic diseases, we are preventing future pandemics.

**Sustainable agriculture**: The veterinary profession can champion sustainable, climate-smart livestock practices. Advising farmers on sustainable livestock management can reduce greenhouse gas emissions, mitigate deforestation, and support food security.

**Biodiversity conservation**: Veterinarians have the knowledge and tools to protect endangered species and promote biodiversity. With climate change contributing to habitat destruction, veterinarians must work closely with conservationists to safeguard wildlife and preserve ecosystems.

**Climate-resilient practices**: Veterinarians can encourage climate resilience within the industry by promoting better practices for animal care in extreme weather conditions, such as heat stress management, disease prevention, and water conservation. These help build sustainable, adaptive systems that protect both livelihoods and ecosystems.

**Community leadership**: Veterinarians are trusted voices in their communities. We can lead awareness campaigns, educate the public on the effects of climate change on animal health that would significantly impact human health, and advocate for policies that promote climate actions at local, national, and international levels.

## The time to act is now

There is no more time for complacency. The worst impacts of climate change are already here, and we must harness the power of veterinary medicine as a tool for climate action and health security. The veterinary profession must be more than reactive; it must be proactive.

As climate advocates, veterinarians can push for systemic changes that support sustainable livestock farming, wildlife conservation, responsible use of natural resources and more robust environmental protection. Moreover, we can lobby for more significant investments in climate resilience research, primarily disease surveillance and prevention.

The urgency is apparent. Each of us has a role to play. It is time to rethink how we educate future veterinarians. Veterinary schools must adapt their curriculum to address climate change. Veterinary practices must work towards reducing their carbon footprints and modelling sustainability. Furthermore, veterinary voices must join the growing chorus demanding climate justice for all species.

## Conclusions

In conclusion, veterinarians have the expertise, the influence, and the moral responsibility to act. As climate change alters our world, veterinarians must become the champions of a vision where animals, humans, and the environment can thrive together. Every veterinarian, veterinary student, and allied professional must become a climate warrior, advocating for sustainable livestock practices, speaking up for animal welfare, pushing for climate-focused education in institutions, joining climate initiatives and lobbying for policy changes that protect the planet. The challenges may seem impossible, but every action counts. Together, we can shape a future where veterinary medicine is not just a bystander but a powerful force for positive change, a profession that not only heals animals but also safeguards the planet for generations to come. In the words of Jane Goodall, “What you do makes a difference, and you have to decide what kind of difference you want to make.” Let us make the difference our world desperately needs.

## Ethics and consent

Ethics and consent not required for this article.

## Data Availability

No data are associated with this article.
