# Supplementary material for: Climate Change and Veterinary Medicine: A Call to Action for a Healthier Planet
Source: F1000Res. 2024 Nov 13;13:1360. [Version 1] doi: 10.12688/f1000research.158307.1 (PMC11724204; doi:10.12688/f1000research.158307.1)
Supplement: Peer review history

# Reviewer report: Reviewer response for version 1

**DOI:** 10.5256/f1000research.173879.r340715

**Published:** 2025-01-10

**Authors:** Robyn Alders

**Affiliations:** 1 Royal Veterinary College, University of London, London, UK; 2 Development Policy Centre, Australian National University,, Canberra,, Australia

This opinion article addresses a crucial topic and the author is to be commended for their efforts. 

 There is growing literature on the topic, and the author has cited only one reference for each of the key points. Given the importance of the topic, it would be ideal to include a second citation for key points.

 The brevity of the opinion means that some points do not receive in-depth attention. For example, there is a large and growing literature on GHG emissions from livestock with some authors questioning the robustness of some data. 

 The Conclusions are valid.  However, it would be ideal to also mention:

 * the importance of animal genetics to animal health. Veterinarians should encourage the raising of animals that are well-adapted to their local environments, requiring fewer inputs such as antimicrobials. See FAO 2025: Benefits from and threats to animal genetic resources; and

 * the need for climate-smart actions to complement actions addressing other planetary boundaries. See WOAH 2021: Planetary Boundaries and Veterinary Services.

 Additional comments:

 * In the Abstract, it is suggested that "As the stewards of animal health" be changed to "As stewards of animal health". Veterinarians are not the only stewards of animal health.

 * Under sustainable agriculture, it would be ideal to mention that improved animal health can contribute to improved nutrient yields by reducing losses of human-edible components of animal carcases, i.e. meat and red and green offal.

Is the topic of the opinion article discussed accurately in the context of the current literature?

Partly

Are arguments sufficiently supported by evidence from the published literature?

Partly

Are all factual statements correct and adequately supported by citations?

Partly

Are the conclusions drawn balanced and justified on the basis of the presented arguments?

Partly

Reviewer Expertise:

One Health, sustainable livestock production, animal health, disease prevention

I confirm that I have read this submission and believe that I have an appropriate level of expertise to confirm that it is of an acceptable scientific standard, however I have significant reservations, as outlined above.

# Reviewer report: Reviewer response for version 1

**DOI:** 10.5256/f1000research.173879.r352442

**Published:** 2024-12-28

**Authors:** Gustavo J Nagy

**Affiliations:** 1 Instituto de Ecología y Ciencias Ambientales (IECA), Universidad de la República (UdelaR), Montevideo, Uruguay

The author analyzed the increasing role of veterinarians in the Environmental and Public Health domains beyond their traditional animal health stewards and safeguards.

 The author focuses on the importance of climate change, highlighting the carbon emissions from livestock and how climate change and the increasing frequency of extreme weather affect animal health by spreading vector-borne diseases.

 The author's strong advocacy for the One Health approach to sustainable agriculture and biodiversity conservation is a key point. This approach places veterinarians' roles at the center of One Health, enabling them to better address animal and planetary health issues beyond the traditional care of livestock.

 The author highlights the urgent need for climate action—both adaptation and mitigation—underscoring the pivotal role of veterinarians in managing it. He presents a list of well-known activities where veterinarians can act, which is not novel and is typically found with the assistance of AI generators.  However, an Opinion Article is not that novel; it should address relevant topics, and this article does it.

 In this vein, the author's emphasis on a new vision of environmental protection, sustainable agriculture, Health, and biodiversity conservation calls for proactive systemic changes. Veterinarians must be proactive instead of reactive to the above mentioned issues and push for systemic changes. He mentions several relevant topics, such as the need for a new approach to teaching veterinary medicine, including carbon management and sustainable practices.

 The author highlights the veterinarian's capacity and responsibility to become a champion regarding the environment and animal and human Health. A One-Health approach, including allied professionals, is crucial to achieving One Health with a Planetary Vision.

 The author makes a common mistake (for non-climate change specialists) by using mitigation and mitigation instead of reduction/reduction, for instance, "mitigate deforestation." In climate change, mitigation refers to the carbon budget (C emission-C sink) and the actions to balance them better.

 Finally, although the citations are correct and relatively new, they should have included a few more recent ones -from 2022 to 2024-.

 Also, as an "Allied scientist" to the One Health approach from the Earth and Environmental Sciences focused on Interdisciplinary Climate studies, I feel the need to punctuate that this Opinion Article about One Health could have put a bit more emphasis on the Planetary Health approach , which I firmly believe merges with One Health, mainly when the focus is on climate change.

Is the topic of the opinion article discussed accurately in the context of the current literature?

Yes

Are arguments sufficiently supported by evidence from the published literature?

Yes

Are all factual statements correct and adequately supported by citations?

Partly

Are the conclusions drawn balanced and justified on the basis of the presented arguments?

Yes

Reviewer Expertise:

Interdisicplinary Climate Studies. Climate Change. Environemntal Sciences. Climate Adaptation. Planetary Health.

I confirm that I have read this submission and believe that I have an appropriate level of expertise to confirm that it is of an acceptable scientific standard.

# Reviewer report: Reviewer response for version 1

**DOI:** 10.5256/f1000research.173879.r344540

**Published:** 2024-12-03

**Authors:** I Made Dwi Mertha Adnyana

**Affiliations:** 1 Department of Biology, Faculty of Information Technology and Science, Universitas Hindu Indonesia, East Denpasar, Indonesia

Dear Authors, 

 The manuscript presents an important and timely perspective on the role of veterinarians in addressing climate change challenges. The manuscript addresses an important topic and provides a good foundation for discussion, but requires substantial revision to strengthen its academic rigor and practical utility.

**Abstract** 

 The first paragraph (lines 6–15) is heavily focused on problem description but lacks quantification of impacts. Specific statistics or concrete examples would strengthen the opening argument. The second paragraph (lines 16–27) discussing action steps is somewhat vague. Phrases like "integrate climate adaptation and mitigation strategies" need more specific examples or a clearer definition of what these strategies entail. The final paragraph (lines 28-36) could be more impactful by including specific, measurable outcomes or goals rather than general statements about building "climate-resilient practices."

**Keywords**

 The keywords (lines 261-280) are appropriate but could be more specific to help with database searches. Consider adding terms related to specific veterinary practices or climate change mitigation strategies.

**Introduction**

 The introduction (lines 154-239) provides a solid foundation but would benefit from more specific data and concrete examples of how climate change is already impacting veterinary practice. While the 14.5% greenhouse gas emission figure from livestock is cited, the introduction should better quantify other key impacts. Additionally, the transition between paragraphs could be improved to create better flow and logical progression of ideas.

**Why Must Veterinary Professionals Act?**

 In lines 689-725, the five key areas of action (zoonotic disease prevention, sustainable agriculture, biodiversity conservation, climate-resilient practices, and community leadership) are well-identified but lack specific implementation strategies. Each area would benefit from concrete examples of successful interventions or pilot programs. The manuscript should provide more detailed guidance on how veterinarians can practically implement these actions in their daily practice.

**The Time to Act Section**

 Lines 154-188 make broad statements about urgency but would be strengthened by including specific timelines, benchmarks, or goals for the veterinary profession. The section lacks measurable objectives that would help guide institutional and individual responses.

**References and Evidence Base** 

 The paper relies heavily on reports and some older references (e.g., Ziswiler 2012). More recent peer-reviewed literature should be incorporated, particularly regarding emerging climate impacts on animal health and innovative veterinary responses (Red 1 and Ref 2). The manuscript would benefit from including case studies or success stories where veterinary interventions have effectively addressed climate-related challenges.

Is the topic of the opinion article discussed accurately in the context of the current literature?

Partly

Are arguments sufficiently supported by evidence from the published literature?

Partly

Are all factual statements correct and adequately supported by citations?

Yes

Are the conclusions drawn balanced and justified on the basis of the presented arguments?

Partly

Reviewer Expertise:

One Health, Epidemiology and Biostatistic, zoonotic disease

I confirm that I have read this submission and believe that I have an appropriate level of expertise to confirm that it is of an acceptable scientific standard, however I have significant reservations, as outlined above.

## References

[1] : Traversed dynamics of climate change and One Health. *Environmental Sciences Europe* .2024;36(1) : 10.1186/s12302-024-00931-8 10.1186/s12302-024-00931-8

[2] : One Health approach and zoonotic diseases in Indonesia: Urgency of implementation and challenges. *Narra J* .2023;3(3) : 10.52225/narra.v3i3.257 e257 10.52225/narra.v3i3.257 38455621 PMC10919696
